# Statin Therapy and Pancreatitis: A Multi-Institutional Retrospective Analysis

**DOI:** 10.7759/cureus.51723

**Published:** 2024-01-05

**Authors:** Caroline E O'Connor, Brittany Q Dang, Brittany Miles, James Mackey

**Affiliations:** 1 Internal Medicine, University of Texas Medical Branch, Galveston, USA; 2 Radiology, Baylor University Medical Center, Dallas, USA; 3 Hematology and Oncology, Baylor University Medical Center, Dallas, USA

**Keywords:** inflammation, lipid-lowering agents, statins, drug-induced pancreatitis, acute pancreatitis

## Abstract

Introduction: Acute pancreatitis is a serious condition that has numerous etiologies and often requires hospital admission due to its high mortality rates. Statins are used worldwide to reduce the risk of cardiovascular disease. Some studies have shown an association between long-term statin use and acute pancreatitis. However, other studies have shown no effect or even postulated a mild protective effect. Due to conflicting information in the medical literature, the relationship between statins and acute pancreatitis remains unclear. The current study uses the TriNetX global research database to further investigate the impact of statin use on the development of acute pancreatitis over a five-year period.

Methods: Two cohorts were created using the TriNetX global research database. One group consisted of patients not taking statins, while the other group included patients taking any statins. Patients in both groups were required to be between the ages of 40 and 75 and had normal low-density lipoprotein cholesterol (LDL) (≤200 mg/dl) and triglyceride (≤150 mg/dl) levels. Patients were matched for age, gender, race, and comorbidities. The statin group was then compared to the no-statin group and measured for the outcome of the incidence of acute pancreatitis and the frequency of episodes within the first five years of statin use. Patients who experienced any acute pancreatitis episode before starting statin therapy or before meeting inclusion criteria were excluded from the study.

Results: Patients on statin therapy were significantly more likely to develop acute pancreatitis compared to patients not taking statin therapy (risk ratio 1.332, 95% CI: 1.242-1.429, P<0.0001). However, the statin group had a lower mean number of pancreatitis episodes than the no-statin group (4.6 vs. 5.3, P=0.043).

Conclusion: The results from this large global dataset support the previously established idea that prolonged use of statins is associated with an increased risk of pancreatitis. Clinicians should strongly consider statin-induced pancreatitis when other common etiologies have been ruled out.

## Introduction

Acute pancreatitis is considered one of the most common gastrointestinal causes of hospitalization in the United States [1]. It is estimated that 210,000 patients are hospitalized annually due to acute pancreatitis [2]. Additionally, acute pancreatitis is associated with an overall mortality rate of approximately five percent [3]. Acute pancreatitis occurs as a result of damage to the acinar cells or injury to the pancreatic duct, leading to pancreatic enzymes accumulating and becoming activated in the pancreas rather than in the gastrointestinal tract [4]. The presence of activated enzymes in the pancreas causes autodigestion of the pancreatic parenchyma and peripancreatic tissue [4]. The resulting inflammatory response leads to an increase in the vascular permeability of the pancreas [5]. This condition can rapidly progress to sepsis, systemic inflammatory response syndrome, organ failure, and mortality if not treated immediately [6,7].

Statins have become increasingly recognized as a cause of drug-induced pancreatitis, accounting for 1.4 to 2 percent of all cases of acute pancreatitis [8], with over 23 cases of acute pancreatitis secondary to statin use having been cited [9]. Statins function to reduce serum cholesterol levels and cardiovascular risk by inhibiting 3-hydroxy-3-methylglutaryl-coenzyme A (HMG-CoA) reductase [10]. From 2018 to 2019, there were approximately 92 million reported statin users in the United States [11]. Drugs with the potential to cause drug-induced pancreatitis are organized into the Badalov classification system [12]. This system takes into account the number of cases reported, re-challenge, and latency period [12]. Drugs in classes I and II have the highest potential for causing acute pancreatitis [12]. For a long period of time, statins were considered class Ia drugs [12]. However, more recently, many statins have started to be classified as either lower-risk class 3b or class 4 due to the increasing number of high-quality case reports with and without re-challenge or clinical latency [13]. Moreover, recent studies have challenged previous associations between statins and acute pancreatitis, claiming no global effect of statins on pancreatitis [14] or even a reduced risk of pancreatitis [15].

Statin-induced pancreatitis is not always recognized or correctly diagnosed in emergency departments and remains an important etiology to consider because the avoidance of causative medications has the potential to prevent initial and future episodes. However, due to the conflicting medical literature, further research is needed to determine whether statins increase the risk of acute pancreatitis. In this study, we used the TriNetX research platform to examine the effects of statin use on acute pancreatitis exacerbations over five years on a global scale.

The study was presented orally at the Society of Academic Emergency Medicine 2023 Annual Meeting on May 18, 2023, under the title "Statin Therapy and Increased Risk of Pancreatitis: An Analysis of 3,691,640 Patients." The abstract was later published in a special issue of their journal, Academic Emergency Medicine, as a result of the presentation. A full original research article of this study has not been published and is not under consideration for any other journal. The current study has been updated to include a larger patient population since the original oral presentation.

## Materials and methods

This study utilized the TriNetX global research database, which grants access to anonymized medical data from over 108 million patients across 72 large healthcare organizations. The patient information used in this study was deidentified and did not involve the collection, use, or transmission of any identifiable patient information.

Two cohorts were created for comparison using the TriNetX platform. Each group consisted of both men and women between the ages of 40 and 75 with normal low-density lipoprotein cholesterol (LDL) (≤200 mg/dl) and triglyceride (≤150 mg/dl) levels. The use of statins was identified by the medication codes for each: atorvastatin (83367), fluvastatin (41127), lovastatin (6472), pitavastatin (861634), pravastatin (42463), rosuvastatin (301542), and simvastatin (36567). One cohort consisted of patients who were not on treatment with any statin, and the second had patients who were on treatment with any of the identified statins. The statin cohort was compared against the cohort that was not on statin therapy. All cohorts were matched for age, gender, race, and familial hypercholesterolemia (E78.01), pure hyperglyceridemia (E78.1), nicotine dependence (F17.21), alcohol dependence (F10.2), other diseases of the biliary tract (K83), obesity (E66), and type 2 diabetes mellitus (E11) using their corresponding International Classification of Disease (ICD-10) codes. Patients who had the outcome of acute pancreatitis before starting statin therapy or before meeting the inclusion criteria were excluded from this study.

They were then measured for the primary outcome of acute pancreatitis (K85) and the secondary outcome of the number of pancreatitis episodes within the first five years of statin use. Propensity score matching through TriNetX was performed using logistic regression analysis and the greedy nearest neighbor algorithm. A 95% confidence interval (95% CI) was used in the calculation of the risk ratio of developing acute pancreatitis within the first five years of statin therapy. The statistical significance of the risk ratio was determined using a p-value (P) threshold of 0.05. A comparison of the mean number of pancreatitis episodes was conducted using a chi-square analysis and a statistical significance threshold of 0.05.

## Results

After propensity score matching and applying exclusion criteria, 233,425 patients in the no-statin group and 233,647 patients in the statin group were included in the final analysis. The no-statin group had an average age of 54.5 years and was composed of 48.8% females and 51.2% males. The statin group had an average age of 53.9 years and was composed of 49.6% females and 50.4% males (Table 1). 

**Table 1:** Baseline demographics and comorbidities of patients not on statin therapy versus patients on statin therapy. Values are presented as mean ± standard deviation or percentage.

Variable	No statin (n=236,895)	Any statin (n=236,895)
Age at index	54.5 ± 9.0	53.9 ± 8.9
Current age	59.9 ± 8.9	59.6 ± 8.8
Female	48.8%	49.6%
Male	51.2%	50.4%
White	65.2%	64.9%
Black or African-American	23.3%	23.3%
Other race	4.2%	4.3%
Hyperlipidemia (E78.5)	26.8%	28.2%
Familial hypercholesterolemia (E78.01)	0.2%	0.2%
Pure hyperglyceridemia (E78.1)	2.0%	2.0%
Nicotine dependence, cigarettes (F17.21)	10.2%	9.9%
Alcohol dependence (F10.2)	4.3%	4.0%
Other disease of the biliary tract (K83)	1.0%	1.0%
Overweight and obesity (E66)	25.4%	25.1%
Type 2 diabetes mellitus (E11)	14.1%	15.9%

It was found that patients on statin therapy were approximately 33% more likely to develop pancreatitis compared to patients not on statin therapy (risk ratio 1.332, 95% CI 1.242-1.429, P<0.0001). Among patients who developed pancreatitis, those not on statin therapy were found to have a significantly greater mean number of pancreatitis episodes (5.3 vs. 4.6, P=0.043) (Table 2).

**Table 2:** Risk analysis of acute pancreatitis in patients not on statin therapy compared to patients on statin therapy.

Variable	No statin (n=233,425)	Statin (n=233,647)
Patients who developed pancreatitis	1,355	1,807
Risk percent	0.58%	0.77%
Mean number of instances	5.29 ± 12.7	4.56 ± 7.4

## Discussion

In the current study of 467,072 patients, those who were on statin therapy had a 33% higher risk of developing acute pancreatitis over a five-year period compared to patients not taking statins (Table 2). Although fewer patients in the no-statin group developed acute pancreatitis, those who did experienced a greater number of episodes compared to the statin cohort (Table 2). The significantly higher risk of developing acute pancreatitis in patients taking a statin is consistent with other studies in the literature evaluating this association [9,12].

While the exact mechanism of statin-induced pancreatitis has not been elucidated, several potential mechanisms have been posited in the literature. Suggested mechanisms include direct cellular toxicity, metabolic effects, an immune-mediated inflammatory response, and pancreatic duct constriction [5]. The onset of statin-induced acute pancreatitis has been seen with a latency period of anywhere from hours to years after the prescription of a statin [4]. This variation makes a mechanism of direct toxic effect or accumulation of a toxic metabolite most likely [4]. This theory allows for the possibility that long-term use of statins may be associated with an increased risk for acute pancreatitis. Another commonly suggested mechanism of statin-induced acute pancreatitis is the interaction of multiple drugs metabolized by CYP3A4 [4]. Reports of acute pancreatitis after starting multi-drug therapy with medications primarily metabolized by CYP4A3, such as simvastatin and venlafaxine together, exist in the literature [8]. This mechanism could explain the difference in the safety profiles of different statins [8]. For example, pravastatin is the only statin not metabolized by CYP3A4, and it is associated with a lower incidence of adverse drug reactions than atorvastatin and other statin medications [8]. The degree to which statins inhibit CYP3A4 and the degree of their lipophilicity may be correlated to their risk of causing acute pancreatitis [16].

Drug-induced pancreatitis is a diagnosis that can only be made after other possible causes of pancreatitis are excluded [4]. The incidence of this kind of pancreatitis is dependent on the accuracy of clinicians in ruling out other causes. However, for patients who have multiple risk factors and comorbidities or who take complex medication regimens, it can be more difficult to diagnose drug-induced pancreatitis. Pancreatitis is a medical condition that can be accurately diagnosed through a combination of clinical presentation and laboratory testing. The diagnosis is typically established by performing serum lipase and amylase tests [4]. However, in cases where the diagnosis is uncertain and requires further investigation, additional tests such as serum trypsinogen and other specific inflammatory markers may be utilized [4].

This study, while providing valuable insights into the association between statin use and drug-induced pancreatitis episodes, has certain limitations that warrant consideration. First, this study was constrained by the inability to quantify or account for the severity of pancreatitis episodes, given the large cohort size and limitations of the TriNetX platform. As such, we are not able to refute the findings of other publications mentioned above that statins could provide an anti-inflammatory, protective factor in limiting the severity of short-term pancreatitis episodes. Furthermore, our study did not encompass an analysis of emergency department visits or the number of hospital admissions related to pancreatitis. These aspects might be of significant interest to those concerned with the associated healthcare system costs. Future research directions could explore the feasibility of utilizing C-reactive protein as a marker for the severity of pancreatitis. In addition, forthcoming investigations could compare specific statin medications to evaluate the relative frequency and severity of pancreatitis episodes in those with high- or low-risk profiles. Further exploration of such factors would help provide a more nuanced understanding of the relationship between statin use and drug-induced pancreatitis.

## Conclusions

Acute pancreatitis is a complex and potentially life-threatening condition characterized by inflammation and the accumulation of pancreatic enzymes. The results from this large global dataset support the idea that the use of statins is associated with an increased risk of acute pancreatitis. The anti-inflammatory hypothesis of decreasing inflammation and decreasing pancreatitis episodes is not supported by this study. Several potential mechanisms underlying statins and pancreatitis have been proposed. Continued research on the mechanism behind statin-induced acute pancreatitis could provide guidance on the best treatment strategies to ensure the safe use of statin medication and improve patient care. Clinicians should strongly consider statin-induced pancreatitis when other common etiologies of acute pancreatitis have been ruled out. Additionally, clinicians should regularly monitor for and educate patients about the signs and symptoms of acute pancreatitis in patients on statin therapy to increase awareness about this potential long-term complication.
